# The Histone Deacetylase Inhibitor AN7, Attenuates Choroidal Neovascularization in a Mouse Model

**DOI:** 10.3390/ijms20030714

**Published:** 2019-02-07

**Authors:** Mor Dahbash, Ruti Sella, Elinor Megiddo-Barnir, Yael Nisgav, Nataly Tarasenko, Dov Weinberger, Ada Rephaeli, Tami Livnat

**Affiliations:** 1 Sackler Faculty of Medicine, Tel Aviv University, Tel Aviv 69978, Israel; mor.dachbash@gmail.com (M.D.); rutibd@gmail.com (R.S.); nataliyt@post.tau.ac.il (N.T.); dwin@zahav.net.il (D.W.); Adarephaeli@gmail.com (A.R.); 2 Laboratory of Eye Research, Felsenstein Medical Research Center, Petach Tikva 49100, Israel; ynisgav@gmail.com; 3 Department of Ophthalmology, Rabin Medical Center, Petach Tikva 49100, Israel; emegiddo@gmail.com; 4 Laboratory of Experimental Pharmacology and Oncology, Felsenstein Medical Research Center, Petach Tikva 49100, Israel; 5 National Hemophilia Center, Institute of Thrombosis, and the Amalia Biron Research Institute of Thrombosis and Hemostasis, Sheba Medical Center, Tel Hashomer 52621, Israel

**Keywords:** AN7, bevacizumab, choroidal neovascularization, histone acetylation, histone deacetylase inhibitor, hypoxia, mouse model, retinal pigmented epithelium, vascular endothelial growth factor

## Abstract

Choroidal neovascularization (CNV) is a complication of age-related macular degeneration and a major contributing factor to vision loss. In this paper, we show that in a mouse model of laser-induced CNV, systemic administration of Butyroyloxymethyl-diethyl phosphate (AN7), a histone deacetylase inhibitor (HDACi), significantly reduced CNV area and vascular leakage, as measured by choroidal flatmounts and fluorescein angiography. CNV area reduction by systemic AN7 treatment was similar to that achieved by intravitreal bevacizumab treatment. The expression of vascular endothelial growth factor (VEGF), fibroblast growth factor (FGF-2), and the endothelial cells marker CD31, was lower in the AN7 treated group in comparison to the control group at the laser lesion site. In vitro, AN7 facilitated retinal pigmented epithelium (RPE) cells tight junctions’ integrity during hypoxia, by protecting the hexagonal pattern of ZO-1 protein in the cell borders, hence reducing RPE permeability. In conclusion, systemic AN7 should be further investigated as a possible effective treatment for CNV.

## 1. Introduction

Choroidal neovascularization (CNV) is the pathological growth of immature blood vessels from the choroid underlying the retinal pigmented epithelium (RPE) towards the sensory retina. It is a complication of age-related macular degeneration (AMD) and a major contributing factor to vision loss. The newly formed blood vessels are immature, lack structural integrity, and leak fluid, leading to hemorrhage and exudates, accompanied by fibrosis [1,2,3,4].

In AMD, angiogenic factors, such as vascular endothelial growth factor (VEGF), are excessively secreted by the RPE layer that forms the outer Blood-Retina Barrier (oBRB), and may contribute to the stimulation of CNV and breakdown of the oBRB [5,6,7].

Histone acetylation status plays a pivotal role in the epigenetic modulation of gene expression [8,9,10]. The status of acetylation is maintained by a dynamic balance between histone acetyl transferases (HATs) and histone deacetylases (HDACs) [11]. HATs add acetyl groups on lysine residues of the histones tails, resulting in the reduction of the electrostatic attraction of the negative backbone of the DNA to the histones, loosening the chromatin structure to a more accessible form and enabling active transcription. In contrast, HDACs remove acetyl groups from lysine residues of the histone tails, leading to a more compact and less accessible chromatin [9,10,11,12].

Data regarding the importance of histone acetylation to the regulation of cell behavior has led to increased interest in the role of HDAC inhibitors (HDACi) as potential pharmacological agents, mainly for cancer prevention and treatment [13,14,15].

The effect that different HDACs have on the eye is yet to be genetically explored. Studies in rodents have shown that the retina expresses HDACs 1 to 6, and that HDACs 1, 2, 3, and 6 may constitute 98% of the total HDAC activity [16,17,18].

While reduced expression of HDACs 1, 2, 5 and 6 was observed in AMD [19], other studies addressing the potential use of HDACi’s in the retina have indicated that HDAC inhibition can protect the retina from acute injury [16,20,21,22,23,24] and may also have an inhibitory effect on CNV development [25,26].

Butyroyloxymethyl-diethyl phosphate (AN7) is a water-soluble and orally bioavailable prodrug of the HDACi butyric acid, and as such, inhibits HDAC classes I and II, which results in the hyperacetylation of histones H3 and H4 [27,28].

AN7 has been shown to effectively stimulate reduction of vascularization in a variety of tissues in vitro, ex vivo, and in vivo [27,28,29,30,31,32]. These previous reports prompted the notion that AN7 may serve as an inhibitor of pathological choroidal angiogenesis as well. We therefore directly evaluated the effect of AN7 on laser-induced CNV using a mouse model and assessed its effect on RPE cell permeability in vitro.

## 2. Results

### 2.1. AN7 Treatment Elevates Histone H3 Acetylation Levels in Laser-induced CNV Lesions

**Figure 1:**
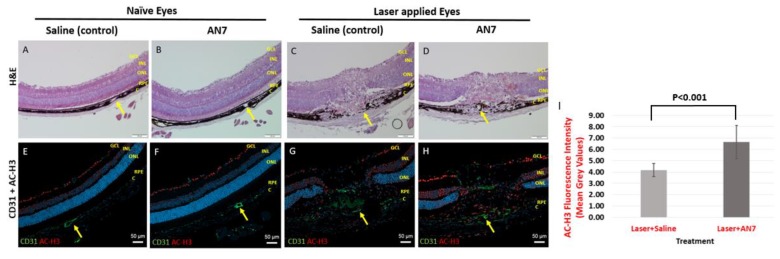
AN7 treatment elevates Histone H3 acetylation levels in laser-applied eyes. Representative images of cryosections from naïve eyes (without laser applications) of mice treated by intraperitoneal (IP) AN7 or saline (A,B,E,F) and lesions sites of laser-applied eyes of mice treated with IP AN7 or saline (C,D,G,H), from day 7 post laser application. (**A**–**D**) Hematoxylin and Eosin (H&E). Scale bar, 100 µm. Yellow arrows mark the same blood vessel in the H&E and the corresponding immunostaining image to allow orientation. (**E**–**H**) Immunostaining for CD31 (green), acetylated histone H3 (AC-H3; red) and cells nuclei, DAPI (blue). GCL, Ganglion Cells Layer; INL, Inner Nuclear Layer; ONL, Outer Nuclear Layer; RPE, Retinal Pigmented Epithelium; C, Choroid. Scale bar, 50 µm. (**I**) Quantification of AC-H3 staining in laser applied eyes.

Figure 1 shows representative images of cryosections stained for Hematoxylin and Eosin (H&E), anti-CD31 and anti- acetylated histone H3 (AC-H3) from naïve eyes (no laser applied) and laser-applied eyes of mice treated with AN7 or saline, from day 7 post laser induction.

In the naïve eyes, AN7 treatment did not induce structural changes of the retina in comparison to control as indicated by H&E images (Figure 1A,B). Moreover, no significant variation in the endothelial cells marker CD31 (green) and AC-H3 (red) staining pattern was noticed between naïve eyes, treated with AN7 in comparison to control (Figure 1E,F). AC-H3 staining was observed mainly in the ganglion cell layer (GCL) showing the basal histone H3 acetylation status.

The effect of intraperitoneal (IP) administration of AN7 on eyes subjected to laser applications is demonstrated in Figure 1C,D,G,H. Representative images of cryosections of lesion sites taken 7 days post laser application show disorganized retinal layers, including disruption of the RPE layer in both saline and AN7-treated eyes. Newly formed blood vessels, stained for CD31, penetrated from the choroid through the sensory retina (Figure 1G,H). AC-H3 staining was observed not only in the GCL, but also in other layers of the retina (i.e., inner nuclear layer, outer nuclear layer, RPE) as well as in the choroid and sclera. However, AC-H3 fluorescence intensity was significantly increased from 4.17 ± 0.58 Mean Grey Values (MGV) in the saline control group to 6.65 ± 1.46 MGV in the AN7-treated group (*p* < 0.001; Figure 1I).

### 2.2. Systemic Administration of AN7 Reduces CNV Area in Choroidal Flatmounts

**Figure 2:**
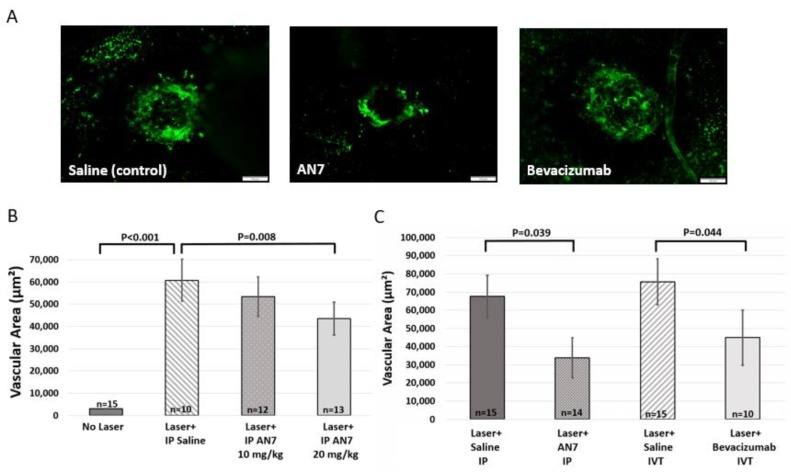
Systemic AN7 treatment reduces choroidal neovascularization (CNV) area. (**A**) Representative images of choroidal flatmounts from day 7 post laser application, with CNV lesions sites from mice treated with saline, AN7 or bevacizumab. Fluorescein isothiocyanate dextran (FITC-dextran) (green) perfused through the blood vessels of the eyes and is seen at the laser lesion site, indicative of CNV formation. Scale bar, 100 µm. (**B**) Quantification of FITC area in choroidal flatmounts (indicative of CNV area) on day 7 from laser photocoagulation. Three laser applications were performed on the right eyes and mice were randomized to intraperitoneal (IP) 20 mg/kg AN7 or 10 mg/kg AN7 or IP saline-control groups, administered immediately following laser photocoagulation and for a total of three times a week thereafter. One-way ANOVA followed by Sidak post hoc test was used for statistical analysis. *n* = number of eyes per group. (**C**) Quantification of FITC area in choroidal flatmounts (indicative of CNV area) on day 7 post laser photocoagulation. Three laser applications were performed on the right eyes. IP injections of AN7 were compared to intravitreal (IVT) injection of bevacizumab and to corresponding saline controls. IP injections of AN7 or saline were administered immediately following laser applications and for a total of three times a week thereafter. IVT injections of bevacizumab or saline were administered once, immediately following the laser applications. One-way ANOVA followed by Sidak post-hoc test was used for statistical analysis. *n* = number of eyes per group.

In order to perform CNV area quantification, 7 days after CNV induction, Fluorescein isothiocyanate dextran (FITC-dextran; green) was perfused and choroidal flatmounts were prepared. Figure 2A shows representative images of laser-induced lesion site from mice treated with saline (control), AN7 or bevacizumab. FITC-dextran perfused from the heart to the blood vessels of the eyes and stained the newly formed blood vessels that penetrated from the choroid towards the retina.

First, a dose dependency experiment was performed (Figure 2B). Significant elevation in vascular area was noticed between eyes without laser applications and eyes with laser applications, confirming the formation of blood vessels, penetrating through the intact black pigmented RPE layer, and indicating CNV (*p* < 0.001, no laser vs. laser and saline).

Treatment with 10 mg/kg AN7 reduced CNV area from 60,751 ± 9327 μm^2^ to 53,319 ± 8941 μm^2^ (nonsignificant), whereas 20 mg/kg AN7 significantly reduced CNV area to 43,527 ± 7350 μm^2^ (*p* = 0.008, laser and saline vs. laser and 20 mg/kg AN7). Consequently, we used AN7 dosage of 20 mg/kg in our in vivo studies.

Next, we compared the efficacy of AN7 to reduce CNV area, to that of bevacizumab, a broadly used medication for neovascular AMD [33]. CNV area was measured by quantification of FITC-dextran area in choroidal flatmounts, prepared on day 7 post laser induction.

Figure 2C shows that IP AN7 reduced CNV area at a similar extent to intravitreal (IVT) bevacizumab. CNV area was significantly reduced from approximately 70,000 μm^2^ in the saline controls, to 33,838 ± 11,057 μm^2^ and 48,472 ± 12,130 μm^2^, by AN7 and bevacizumab, respectively (*p* < 0.05), thus indicating the anti-angiogenic effect of systemic AN7.

We further extended our evaluation and tested the therapeutic potential of oral administration of AN7 (Figure S1). Similar to IP AN7 treatment, oral AN7 treatment significantly reduced CNV area from approximately 60,000 μm^2^ in the saline controls, to 43,527 ± 7350 μm^2^ and 44,002 ± 11,662 μm^2^, by IP AN7 and oral AN7, respectively (*p* < 0.05).

### 2.3. AN7 Reduces CD31, VEGF, and FGF-2 at the Laser Lesion Site

To elucidate the mechanism of AN7 leading to CNV attenuation, we examined the effect of AN7 on the expression of VEGF and fibroblast growth factor (FGF-2), on day 3 post CNV induction, while they were highly expressed [34,35].

**Figure 3:**
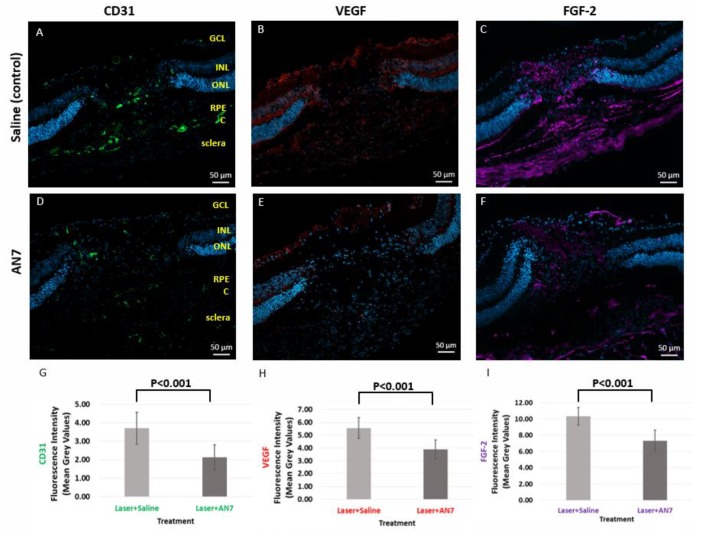
AN7 treatment reduces CD31, vascular endothelial growth factor (VEGF) and fibroblast growth factor 2 (FGF-2). Representative images of laser lesion sites from mice treated with IP 20 mg/kg AN7 or saline (control), from day 3 post laser photocoagulation. Sequential cryosections are stained for endothelial cells marker CD31 (green; **A**,**D**), VEGF (red; **B**,**E**), and FGF-2 (purple; **C**,**F**). Cells nuclei are stained with DAPI (blue). GCL, Ganglion Cells Layer; INL, Inner Nuclear Layer; ONL, Outer Nuclear Layer; RPE, Retinal Pigmented Epithelium; C, Choroid. Scale bar, 50 µm. (**G**) Quantification of CD31 staining in laser applied eyes. (**H**) Quantification of VEGF staining in laser applied eyes. (**I**) Quantification of FGF-2 staining in laser applied eyes.

Figure 3 demonstrates that VEGF (red) and FGF-2 (purple) staining was less prominent at the lesion site of IP AN7 treatment in comparison to control. VEGF fluorescence intensity was significantly decreased from 5.55 ± 0.83 MGV in the saline control group to 3.88 ± 0.74 MGV in the AN7-treated group (*p* < 0.001). FGF-2 fluorescence intensity was significantly decreased from 10.35 ± 1.09 MGV in the saline control group to 7.31 ± 1.33 MGV in the AN7-treated group (*p* < 0.001). These results are in accordance with the statistically significant reduction in CD31 induced by AN7 from 3.7 ± 0.87 MGV in the saline control group to 2.12 ± 0.68 MGV in the AN7-treated group (*p* < 0.001); the less angiogenic factors were expressed, the less endothelial cells were present, showing the anti-angiogenic effect of AN7.

### 2.4. AN7 Reduces Vascular Leakage from CNV Lesions

**Figure 4:**
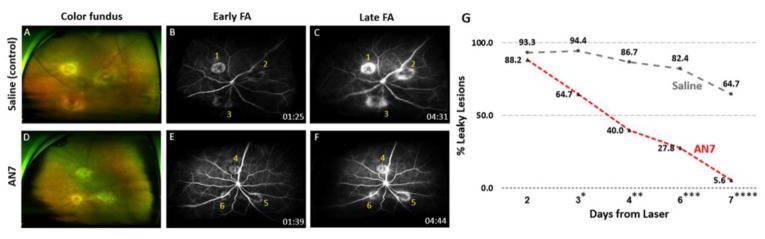
AN7 treatment reduces fluorescein leakage from laser lesions. Representative images of color fundus (**A**,**D**) and fluorescein angiography (FA; **B**,**C**,**E**,**F**) from day 7 post CNV induction of mice treated by intraperitoneal (IP) 20 mg/kg AN7 or saline. Time of imaging post fluorescein injection is indicated at the bottom right side of each angiogram. (**B**,**C**) Early (1 to 2 min post fluorescein injection) and late (4 to 5 min post fluorescein injection) angiograms of an eye from IP saline-control group. Lesion one was classified as stained, while lesions two and three were classified as leaky. (**E**,**F**) Early and late angiograms of an eye from IP 20 mg/kg AN7-treated group. All three lesions were classified as stained. (**G**) The percentage of leaky lesions of total lesions (six mice with 18 lesions total for each group) on days 2 to 7 post CNV induction (the proportions of leaky and stained lesions in each group were tested using the Fisher’s exact test; *Day 3, *p* = 0.041; **Day 4, *p* = 0.021; ***Day 6, *p* = 0.002; ****Day 7, *p* = 0.0003).

Fluorescein angiography (FA) was used in order to detect the effect of AN7 on CNV leakage. Figure 4 shows representative images of *en face* color images of the fundus (Figure 4A,D) and FA, performed on day 7 after CNV induction, revealing three laser spots around the optic disc of each eye. While saline treated eyes demonstrated blurred margins increasing in size over time, in AN7 treated eyes the lesion margins remained stable and distinct over time.

Figure 4B,C of the saline-control demonstrates three hyperfluorescent lesions. Lesions two and three were classified as “leakage”, while lesion one had distinct margins and was therefore classified as “staining”.

Figure 4E,F of the AN7 treatment shows three lesions that were classified as “staining”. Figure 4G summarizes the percentage of leaky lesions of total lesions from day 2 to day 7 post CNV induction. A statistically significant reduction in leaky lesions was observed in the AN7 treatment group, as early as day 3 post CNV induction (*p* = 0.041; Day 3). On day 7 post CNV induction, nearly all lesions of AN7 treatment became stained (17 stained, one leaky; 5.6% leaky lesions), while the saline lesions remained mostly leaking. Some of these became stained as part of the natural course of the healing process (six stained, 11 leaky; 64.7% leaky lesions) (*p* = 0.0003; Day 7).

### 2.5. AN7 Treatment Stabilizes Tight Junctions and Reduces Permeability of RPE Cells Exposed to Hypoxia

To test the effect of AN7 on the oBRB formed by the RPE cells, we used a hypoxia model, as hypoxia is known to induce the expression of angiogenic factors such as VEGF [36]. RPE cells were exposed to 24 h normoxic or hypoxic conditions in the presence or absence of AN7 in the media.

**Figure 5:**
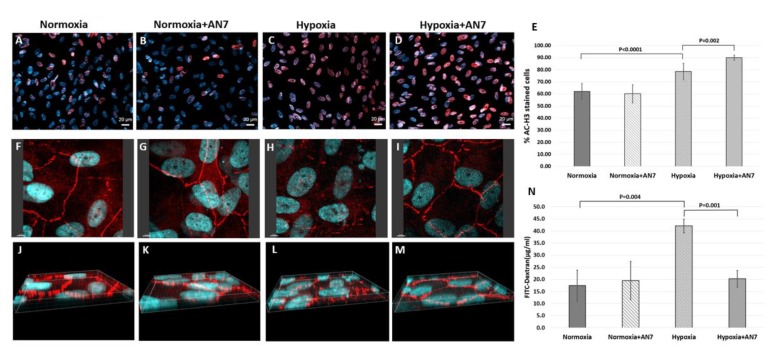
AN7 treatment stabilizes the retinal pigmented epithelium (RPE) monolayer during hypoxia. RPE cells were grown in normoxic or hypoxic conditions for 24 h, in the presence or absence of AN7 in the media. (**A**–**D**) Representative images of RPE cells stained for acetylated histone H3 (AC-H3; red) and cells nuclei, DAPI (blue). Scale bar, 20µm. (**E**) Quantification of the percentage of AC-H3 stained cells of total cells, comparing normoxic and hypoxic conditions in the presence or absence of AN7 in the cell media. One-way ANOVA followed by Tukey’s multiple comparisons test was used for statistical analysis. (**F**–**M**) Representative images of RPE cells stained for the tight junctions associated protein, Zonula Occludens-1 (ZO-1; red) and cells nuclei, DAPI (blue). Scale bar, 5µm. (**E**–**H**) *Z*-axis images are showing the distribution pattern of ZO-1 through the borders of the cells. (**N**) Quantification of FITC-dextran leakage through the RPE cell layer grown on Polyethylene Terephthalate (PET) membranes, comparing normoxic and hypoxic conditions in the presence or absence of AN7 in the cell media. One-way ANOVA followed by Tukey’s multiple comparisons test was used for statistical analysis.

First, AC-H3 staining (Red; Figure 5A–E) was used to validate the selective activity of AN7 on stimulated cells, i.e., exposed to hypoxia. In normoxic conditions (Figure 5A,B), the addition of AN7 did not affect the acetylation status. In hypoxic conditions (Figure 5C,D), however, the addition of AN7 induced a significant elevation in the histone acetylation levels (*p* = 0.002).

Figure 5F to M shows representative images of the tight junctions (TJ)-associated protein Zonula Occludens-1 (ZO-1) staining. Under normoxic conditions, ZO-1 staining was observed partly inside the cells, but mostly at the cell borders (Figure 5F,J), confirming the normal hexagonal shape of RPE cells. Addition of AN7 under normoxic conditions did not affect the ZO-1 localization (Figure 5G,K). In contrast, exposure of RPE cells to hypoxia significantly reduced ZO-1 expression at the cell borders (Figure 5H,L), resulting in the loss of the normal tiling pattern. However, addition of AN7 to the cells media while exposing them to hypoxia protected the normal hexagonal tiling pattern of ZO-1 at the cell borders (Figure 5I,M), indicating the stabilizing effect of AN7 on the TJ of RPE during hypoxia. These results are in correlation with the effect of AN7 on AC-H3 staining during hypoxia.

Figure 5N shows the quantification of FITC-dextran leakage through RPE cell layer. In normoxia, FITC-dextran concentration in the lower chamber was 17.4 μg/mL and the addition of AN7 did not induce any significant effect (19.4 μg/mL). Exposure of RPE to hypoxia, increased FITC-dextran leakage to 42 μg/mL (*p* = 0.004; normoxia vs. hypoxia), indicating the significant elevation in RPE cells permeability. Notably, adding AN7 during hypoxia restored FITC-dextran leakage to normal levels (20.3 μg/mL; *p* = 0.001, hypoxia vs. hypoxia and AN7).

## 3. Discussion

While inhibition of VEGF remains the mainstay of research focus for CNV therapies, anti-VEGF agents do not alleviate the disease or stop its progression in all cases, thus indicating the involvement of additional pathways in chorioretinal angiogenesis. Moreover, repeated intravitreal injections are required for successful treatment, increasing the risk for vision-threatening complications, as the injected eye is exposed to infection, inflammation, intraocular pressure elevation, vitreous hemorrhage, cataract, and retinal detachment, with a consequent loss of vision or loss of eye [37,38,39,40]. Therefore, additional therapeutic agents and alternative routes of administration should be explored.

HDACi’s have been extensively studied in cancer biology and have been found to affect key events in tumor progression by inhibiting proliferation and inducing differentiation and apoptosis in vitro and in vivo [28,41]. Several compounds with HDAC inhibitory activity have been identified, differing in structure, HDAC enzymes specificity, potency, and toxicity. The approval of HDACi’s such as vorinostat, romidepsin, belinostat, and panobinostat, has revolutionized the way cancers are being treated [42]. However, lack of response and development of resistance to the treatment is an issue [43,44]. Improving the selectivity of HDACi’s to amplify their accumulation in cancer cells at a lower dose and thereby reduce the toxic effect [45,46,47] of these drugs on normal healthy cells entail future studies [48].

HDACi’s are currently being evaluated for the treatment of various eye diseases, including retinal degenerative diseases [49]. Studies in rodents have shown that HDACi’s can significantly reduce retinal injury initiated by ischemia/reperfusion [16,24], reduce inflammation in dry eye disease [50], and inhibit postoperative conjunctival fibrosis [51]. Moreover, valproic acid, an HDACi, has been used for treating patients with retinitis pigmentosa [52].

The potential role of HDACi’s in the regulation of CNV formation was previously studied [16,24,26]. Using the laser induced CNV experimental mice model, Chan et al. [25] showed that Trichostatin A (TSA), an HDACi, attenuated CNV formation. However, TSA’s production is costly and highly inefficient [53,54], and TSA studies are considered difficult to reproduce, and its function is largely dependent on the presence or absence of a carrier protein [55]. Thus, TSA is considered mainly a reference substance utilized in the search for new and more efficient HDACi’s.

AN7 has previously been reported to exhibit high selectivity, low toxicity, and significant anti-angiogenic and anti-metastatic properties in different cell lines in vitro [30,41,56] and in various tumors in vivo [30,41,56,57]. It has been suggested that AN7 specifically targets the elevated HDACs activities and expression in cancer cells. The specific suppression of HDACs activity and expression, as well as the difference in the inherent HDAC activity in different cell types was attributed to the anti-cancer efficacy and the selectivity of AN7 [28,41,58]. The well-established anti-angiogenic activities of AN7 [30,56], in addition to the similarity between the angiogenic processes involved in cancer, and the pathologic blood vessels development in the eye, encouraged us to evaluate the protective effect of AN7 in ocular pathologies.

Rephaeli et al. showed that the acute LD_50_ doses of AN7 in C57BL mice were >1 g/kg for IP and >1.2 g/kg for oral administration [27,41], while the anti-metastatic activity of AN7 when administered by either route reached a plateau between 20 to 50 mg/kg and higher doses did not result in any increased activity [28]. Our group previously showed that IP administration of 20 mg/kg AN7 attenuated chemical-burn-induced corneal neovascularization in mice [32], demonstrating the anti-angiogenic effect of systemic AN7 in an ocular pathology. Based on these studies, we speculated that a dosage of 20 mg/kg AN7 would be sufficiently efficacious in treating CNV in a mouse model, and therefore our dose dependency study was performed with a maximal dosage of 20 mg/kg AN7.

Histone acetylation and deacetylation play an important role in transcription modulation [59,60]. The protective effects of AN7 are known to be facilitated by changes in histone acetylation status [27,28]. We demonstrated that both laser induction of CNV in vivo and hypoxia induction in vitro altered histone acetylation status. Treatment of laser-induced CNV with AN7 and the addition of AN7 to RPE cells under hypoxia, both resulted in a statistically significant increase in histone hyperacetylation.

Our in vivo study revealed that AN7 has inhibitory effects on both formation and leakage of pathologic blood vessels, emphasizing the anti-angiogenic effects of AN7 treatment. We have demonstrated that systemic administration of AN7 induced changes in the histone acetylation levels in the retina, leading to VEGF and FGF-2 reduction in the laser lesion area. This is highly important, as these factors are known to be principal in the pathologic neovascularization process [61,62]. These results are in correlation with the observed reduction in the endothelial cells marker CD31 staining, supporting the contribution of angiogenic factors to CNV development, and the involvement of AN7 in attenuating CNV through down regulation of pro-angiogenic factors. However, the exact mechanism by which AN7 reduces CNV is yet to be elucidated. Moreover, it would be interesting to study the combination of AN7 treatment with the conventional bevacizumab treatment, as we have shown that they each have comparable effects on CNV area reduction.

Loss of vision is a main outcome of the newly formed blood vessels being immature and leaky [1,2,3]. Systemic administration of AN7 suppressed fluorescein leakage from CNV lesions. It is likely that the observed reduction in CNV leakage, exerted by AN7 treatment, is due to the significant decrease in CNV lesion area, as well as the reduction in vascular permeability, manifested through the suppression of VEGF and FGF-2.

RPE permeability is known to be increased by VEGF through the functional disruption of TJ proteins, such as ZO-1, leading to destabilization of the oBRB [6,7,63,64]. We have found that AN7 had a protective effect over the barrier features maintained by the RPE, as AN7 protected the hexagonal tiling pattern of ZO-1 and significantly reduced RPE permeability during hypoxia. To the best of our knowledge, this is the first study to show the potential of AN7 as a stabilizer of epithelial barriers in general, and the oBRB of the eye in particular, through TJ modifications.

No less important, our results show no significant deleterious effects of systemic AN7 in naïve eyes or non-stimulated cells. This may be significant for its future safety as a potential drug. Its systemic way of administration may appeal to clinicians who currently rely solely on treatment with intraocular injections.

In conclusion, based on the anti-angiogenic and anti-permeability effects exerted by the systemic administration of AN7 on laser-induced CNV in a mouse model, we propose that AN7 and possibly other low-toxicity, orally-bioavailable HDACi’s, should be considered as potential future candidates for the treatment of persistent, recurrent, or refractory CNV, which requires further investigation.

## 4. Materials and Methods

### 4.1. Animals

Eight-week old male C57BL/6J mice (Envigo RMS, Jerusalem, Israel) weighing 19 to 25 grams, were obtained and handled according to the guidelines of the Association for Research in Vision and Ophthalmology (ARVO) statement for the Use of Animals in Ophthalmic and Visual Research and the approval of the Institutional Animal Care and Use Committee of Rabin Medical Center (Project identification #022-b11353 041116, 4 November 2016).

Mice were anesthetized with an IP injection of ketamine (40 mg/kg; Vetoquinol, Lure, France) and xylazine (10 mg/kg; Eurovet Animal Health BV, Bladel, Netherlands), supplemented with topical anesthesia with oxybuprocaine hydrochloride (0.4%; Fischer Pharmaceutical Labs Ltd., Bnei Brak, Israel) and their pupils were dilated with topical administration of 0.8% tropicamide eye drops (Fischer Pharmaceutical Labs Ltd.).

CNV was performed as previously described by Weinberger et al. [65] Briefly, diode laser indirect ophthalmoscope (Iris Medical Oculight SLX System©, Iridex, Mountain View, CA, USA) was used with laser power of 350 mW for duration of 100 msec, and a condensing lens of 90 diopters. Three laser applications were applied on the right eye, at a distance of 1 to 2 optic disc diameters around the optic nerve. Disruption of the Bruch’s membrane was identified by the appearance of a white bubble at the site of photocoagulation.

Mice were then randomized and treated with AN7 or saline. AN7 was prepared as previously described [27]. Net AN7 is a liquid containing 1 mg/µL at a concentration of 3.93M prior to further dilution. The structure and metabolic products of AN7 are shown in Table 1.

AN7 was formulated in sterile saline just before administration. AN7 (20 mg/kg) or saline (as control) was administered IP immediately following CNV induction and for a total of three times a week. Naïve mice, without laser applications, received IP 20 mg/kg AN7 or saline, three times a week.

In order to compare the efficacy of AN7 with bevacizumab (25 mg/0.1 mL Avastin; Genentech, South San Francisco, CA, USA and Roche, Basel, Switzerland), mice were randomized to 4 groups: AN7 IP injections, saline IP injections, bevacizumab IVT injection, or saline IVT injection.

IVT injections were administered once, immediately post CNV induction, and performed under an operating microscope (Zeiss Opmi Microscope; Carl Zeiss Microscopy GmbH, Jena, Germany). Briefly, a microsyringe (33-gauge; Hamilton) was placed intravitreally in the retrolental space of the right eye, and 1 μL of bevacizumab or saline (as control) were injected. AN7 and saline IP injections were administered three times a week, as described above.

### 4.2. Histology and Immunofluorescence Staining

On days 3 or 7 post CNV induction and treatment initiation with IP 20 mg/kg AN7 or saline, mice were sacrificed by cervical dislocation (*n* = 5 mice per group, total of 10 mice per day 3 receiving one IP injection, and 10 mice per day 7 receiving three IP injections). Eyes were fixed in 4% paraformaldehyde (PFA) for 2 h, washed with phosphate buffered saline (PBS) and gradually incubated with sucrose to a final concentration of 30% sucrose overnight. Eyes were then embedded in OCT compound (Sakura Finetek, Tokyo, Japan) on dry ice and kept in −80 °C. Serial sections of 10 μm thickness were cut using a cryostat (Leica Biosystems, Wetzlar, Germany).

Day 7 cryosections were blocked with 10% normal donkey serum (NDS) and incubated with rat anti-mouse CD31 antibody (BD biosciences, San Jose, CA, USA) and rabbit anti-acetylated histone H3 (Cell Signaling Technology, Danvers, MA, USA). The cryosections were then incubated with the appropriate secondary antibodies (Alexa Fluor488 for CD31 and Alexa Fluor568 for AC-H3; Invitrogen, Carlsbad, CA, USA). Finally, Nuclei were counterstained with DAPI.

Sequential cryosections with comparing regions were stained for H&E (ScyTek Laboratories inc., Logan, UT, USA). Day 3 cryosections were stained using the same immunostaining protocol described above, with rabbit anti-mouse VEGF antibody or rabbit anti-mouse FGF-2 antibody (Abcam plc., Cambridge, UK), and with rat anti-mouse CD31 antibody, followed by the appropriate secondary antibodies (Alexa Fluor568 for VEGF or FGF-2, and Alexa Fluor488 for CD31; Invitrogen, Carlsbad, CA, USA). Images were captured using a fluorescence microscope (Olympus Optical Co., Tokyo, Japan; or Axio Imager.Z2, Carl Zeiss Microscopy GmbH, Jena, Germany).

MGV were used to define AC-H3, VEGF, and FGF-2 fluorescence intensities using ImageJ software (Version 1.51J, NIH, MD, USA). Six slides containing laser lesions sites from 5 eyes of each group (total of 30 slides per group) were used for the aforementioned analysis.

### 4.3. Choroidal Flatmounts and CNV Area Quantification

Seven days post CNV induction and treatment with IP 20 mg/kg AN7, IP saline, IVT bevacizumab or IVT saline, mice were anesthetized (54 mice, *n* = 10 to 15 per group). FITC-dextran (MW = 500 kD, Sigma Aldrich, Rehovot, Israel), diluted in saline to a concentration of 25 mg/mL, was perfused through the mice hearts, and 5 min later mice were euthanized by cervical dislocation.

Eyes were enucleated and fixed in 4% PFA for 2 h. Eyes were then washed with PBS, and the RPE-choroid-sclera complex was carefully isolated, flattened by radial incisions, resulting in choroidal flatmounts, and placed on slides.

Sensory retina flatmounts were used to validate FITC-dextran perfusion from the heart to the blood vessels of the eyes. Therefore, mice without staining on retinal flatmounts were excluded from further analysis.

Images of choroidal and retinal flatmounts were captured using a fluorescence microscope (Olympus Optical Co., Tokyo, Japan). Choroidal flatmounts images were also captured with a light microscope, to eliminate areas transparent to light.

ImageJ software (Version 1.51J, NIH, MD, USA) was used to delineate and quantify FITC-dextran area (indicative of CNV area) on the choroidal flatmounts.

### 4.4. Fluorescein Angiography

Mice with laser induction of CNV were treated with 20 mg/kg AN7 or saline (n = 6 in each group), administered IP immediately following laser application for a total of three times a week (on days 0, 2, 4 post CNV induction). On days 2 to 7 post CNV induction, mice were anesthetized, their pupils dilated, and 0.1 ml 2.5% fluorescein sodium (Novartis, Basel, Switzerland) was injected IP. Vascular leakage was evaluated using the Optos California UWF imaging system (Optos Inc., Marlborough, MA, USA). Color fundus images were also taken.

Two masked retina specialists, not involved in laser photocoagulation or FA imaging, interpreted the fluorescein angiograms. Each laser spot was classified to “Leakage”: Hyperfluorescence gradually increases in intensity over time, with the borders of hyperfluorescence becoming increasingly blurred on the late phases of imaging; or “Staining”: Hyperfluorescence gradually increases in intensity over time, but the borders remain fixed throughout the angiograms.

### 4.5. Cell Culture

Human RPE cells (ARPE-19; ATCC, Manassas, VA, USA) were grown in Dulbecco’s Modified Eagle Medium (DMEM) and Ham’s nutrient mixture F12 (F12; Biological Industries, Beit Ha’emek, Israel) culture medium, containing 10% Fetal Bovine Serum (FBS; Biological Industries, Beit Ha’emek, Israel), 1 mM Glutamine, and 100 U/mL Penicillin, 0.1 mg/mL Streptomycin, 12.5 U/mL Nystatin (PSN; Biological Industries, Beit Ha’emek, Israel) as full medium. Cells were cultured in humidified incubator at 37 °C and 5% CO_2_, for 3 to 4 weeks (passages 10 to 20) to achieve epithelial apical-basal polarity, with culture media being changed every 3 to 4 days.

RPE cells (passages 10 to 20) were grown in normoxic conditions as described above. Hypoxic conditions were provided using hypoxia chamber, with flushes of 1% O_2_ and 5% CO_2_ every 3 h.

### 4.6. Immunofluorescence for RPE Cell Culture

RPE cells were cultured on slides in full medium containing or lacking 100μM AN7, while exposed to normoxic or hypoxic conditions (described above) for 24 h. Slides were then fixed in 4% PFA and blocked with 10% NDS for 1 h, followed by incubation with primary antibodies: Rabbit anti-ZO-1 (Invitrogen, Carlsbad, CA, USA) or rabbit anti-AC-H3; at 4 °C overnight. Alexa Fluor568 was used as secondary antibody. Finally, Nuclei were counterstained with DAPI. 

ZO-1 staining was digitally captured using a confocal microscope (Leica TCS SP8, Leica Biosystems, Wetzlar, Germany), and 3-dimensional images were represented using Imaris software (Version 9.1, Oxford Instruments, Abingdon, UK).

AC-H3 staining was digitally imaged using a fluorescence microscope (Axio Imager.Z2, Carl Zeiss Microscopy GmbH, Jena, Germany), and was quantified using ImageJ software (*n* = 9 images per group) (Version 1.51J, NIH, MD, USA).

### 4.7. RPE Cell Culture Permeability Assay

RPE cells were grown on top of a 1 µM Polyethylene Terephthalate (PET) hanging cell culture inserts (Merck Millipore, Burlington, MA, USA) in 24 wells plate. On the day of experiment, plates were cultured in full medium containing or lacking 100 μM AN7, while exposed to normoxic or hypoxic conditions for 24 h (*n* = 3 wells per group).

Media were discarded, cells were washed with basal medium (DMEM/F12 only), and basal medium was applied to the lower chamber of the inserts. Basal medium containing 1000 µg/mL FITC-dextran (MW = 10 kD, Sigma Aldrich, Rehovot, Israel) was instilled in the upper chamber of the inserts. Fluorescence of media from the lower chambers, representing flow across the RPE cell layer, was measured half an hour following incubation, using the Synergy HT microplate reader (excitation = 485 nm, emission = 528 nm; BioTek Instruments Inc., Winooski, VT, USA). Fluorescence of samples was compared to a calibration curve for the calculation of the FITC-dextran concentration.

### 4.8. Statistical Analysis

Unless indicated otherwise, data are expressed as mean±SD. Statistical analyses were performed (GraphPad Prism 7, CA, USA) using the unpaired *t*-test or the one-way ANOVA, or the Fisher’s exact test. *p* < 0.05 was considered statistically significant.

## Figures and Tables

**Table 1:** Chemical structure and metabolites of AN7.

Name	Structure	Metabolites
AN7 (Butyroyloxymethyl-diethyl phosphate)	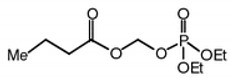	Butyric acid
		Formaldehyde
		Phosphoric acid ethanol

## Supplementary Materials

Supplementary materials can be found at http://www.mdpi.com/1422-0067/20/3/714/s1.
