# Supplementary material for: The Histone Deacetylase Inhibitor AN7, Attenuates Choroidal Neovascularization in a Mouse Model
Source: Int J Mol Sci. 2019 Feb 7;20(3):714. doi: 10.3390/ijms20030714 (PMC6387404; doi:10.3390/ijms20030714)
Supplement: Supplementary file 1 [file ijms-20-00714-s001.pdf]

The Histone Deacetylase Inhibitor AN7, Attenuates Choroidal Neovascularization in a Mouse Model

Supplementary Data

Oral treatment with AN7

Following laser photocoagulation, mice were randomized to 4 groups: intraperitoneal (IP) 20 mg/kg AN7, IP saline, oral 20 mg/kg AN7 or oral saline. Treatment was given immediately following CNV induction and for a total of thrice weekly thereafter. Oral administration was performed with gavage. Choroidal flatmounts analysis was performed on day 7 post laser induction of CNV.

Supplementary Figure S2 shows that both IP and oral treatment with 20 mg/kg AN7 significantly reduced CNV area. IP AN7 significantly reduced CNV area from 60,751±9,327μm^2 to 43,527±7,350μm^2 ($p=0.008$), and oral AN7 significantly reduced CNV area from 58,796±10,812μm^2 to 44,002±11,662μm^2 ($p=0.03$).

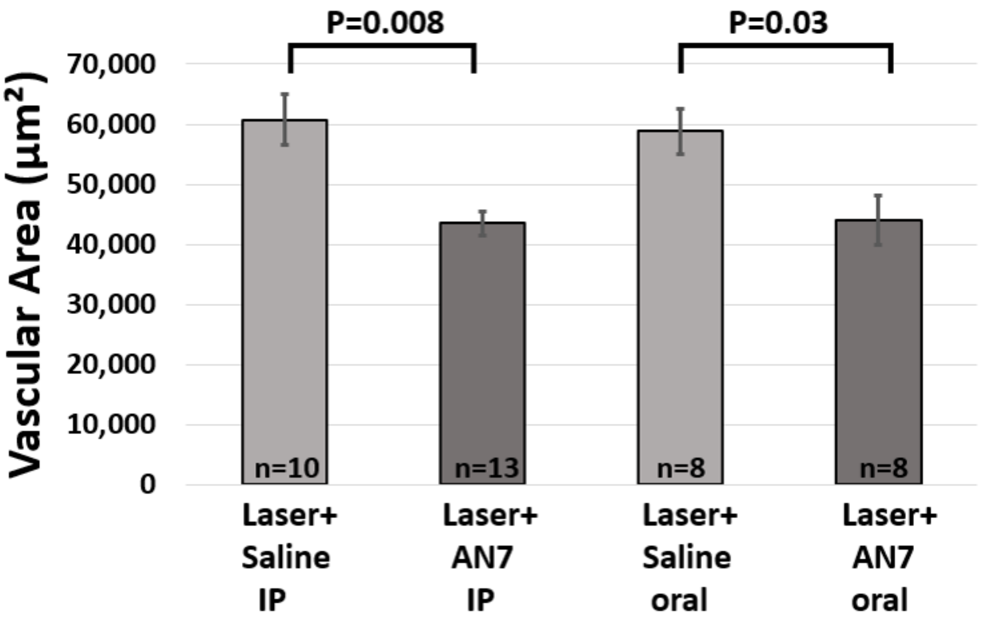

Figure S1. Oral AN7 treatment reduces CNV area.

Quantification of FITC area in choroidal flatmounts (indicative of CNV area) on day 7 post laser photocoagulation. Three laser applications were performed on the right eyes. Intraperitoneal (IP) injections of AN7 was compared to oral administration of AN7 and to corresponding saline controls. 1-way ANOVA followed by Sidak post hoc test was used for statistical analysis. n= number of eyes per group.
